# Dynamics of Fibril Collagen Remodeling by Tumor Cells: A Model of Tumor-Associated Collagen Signatures

**DOI:** 10.3390/cells12232688

**Published:** 2023-11-22

**Authors:** Sharan Poonja, Ana Forero Pinto, Mark C. Lloyd, Mehdi Damaghi, Katarzyna A. Rejniak

**Affiliations:** 1 Integrated Mathematical Oncology Department, H. Lee Moffitt Cancer Center, Research Institute, Tampa, FL 33612, USA; 2 Cancer Biology PhD Program, University of South Florida, Tampa, FL 33612, USA; 3 Fujifilm Healthcare US, Inc., Lexington, MA 02421, USA; mark.lloyd@fujifilm.com; 4 Department of Pathology, Renaissance School of Medicine, Stony Brook University, Stony Brook, NY 11794, USA; 5 Department of Oncologic Sciences, Morsani School of Medicine, University of South Florida, Tampa, FL 33612, USA

**Keywords:** extracellular matrix (ECM), ECM fibril patterns, Tumor-Associated Collagen Signature (TACS), agent-based models, in silico modeling, MultiCell-LF model, tumor microenvironment, tumor-ECM interactions

## Abstract

Many solid tumors are characterized by a dense extracellular matrix (ECM) composed of various ECM fibril proteins. These proteins provide structural support and a biological context for the residing cells. The reciprocal interactions between growing and migrating tumor cells and the surrounding stroma result in dynamic changes in the ECM architecture and its properties. With the use of advanced imaging techniques, several specific patterns in the collagen surrounding the breast tumor have been identified in both tumor murine models and clinical histology images. These tumor-associated collagen signatures (TACS) include loosely organized fibrils far from the tumor and fibrils aligned either parallel or perpendicular to tumor colonies. They are correlated with tumor behavior, such as benign growth or invasive migration. However, it is not fully understood how one specific fibril pattern can be dynamically remodeled to form another alignment. Here, we present a novel multi-cellular lattice-free (*MultiCell-LF*) agent-based model of ECM that, in contrast to static histology images, can simulate dynamic changes between TACSs. This model allowed us to identify the rules of cell–ECM physical interplay and feedback that guided the emergence and transition among various TACSs.

## 1. Introduction

In vivo tumor microenvironments are complex and dynamically changing. The extracellular matrix (ECM), which fills the space between the tumor and stromal cells, is composed of about 300 different proteins [1,2]. The most abundant of these belong to a class of fibrous proteins (e.g., collagens, fibronectins, elastins, or laminins), which are assembled into well-organized meshes and form structural support for the residing cells [3]. ECM organization can be affected by four main processes: deposition, molecular changes, degradation, and physical remodeling [4]. ECM proteins are locally secreted by stromal cells, such as fibroblasts, and their excessive deposition (fibrosis) is often a sign of an aggressive tumor [3,4]. Elevated ECM fibril crosslinking can lead to the formation of prominent fiber bundles and has been associated with increased integrin signaling and tumor progression [5]. Degradation of ECM density and fibril cleavage is a result of the proteolytic activity of various matrix metalloproteinases (MMPs) [6]. These are secreted primarily by stromal cells and are involved in cancer cell invasive migration, the degradation of vascular endothelium during intravasation, and the establishment of new metastatic colonies. Finally, physical ECM remodeling by growing or migrating cells is mediated by pushing or pulling forces and results in ECM fibril alignment, increased density, and modified ECM rigidity. This last type of cell–ECM interaction is the subject of our computational studies. We also focus on collagen, which can be observed in tissue histology slices stained with hematoxylin and eosin (H&E) and can be visualized using second-harmonic generation (SGH) microscopy [7,8].

**Figure 1:**
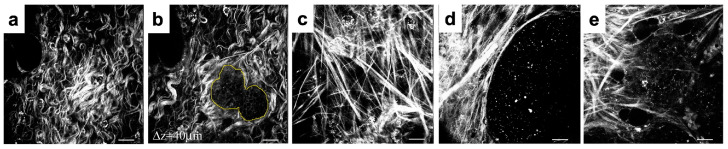
Examples of ECM fibril patterns in mammary tumors in mice. Tumor Associated Collagen Signatures (TACS): (**a**,**b**): TACS-1; (**c,d**): TACS-2; (**e**): TACS-3; adjusted based on [13] and reprinted with permission.

It has been shown that elevated collagen density and fibril alignment can regulate cancer cell signaling, proliferation, polarity, and migration [9,10]. This has been associated with tumor cell invasive potential, metastatic spread, and increased mortality in patients with breast cancers [7,11]. In several experimental studies of breast cancers in mice, specific patterns of collagen fibrils called tumor-associated collagen signatures (TACS) were observed [12,13,14,15]. In particular, the TACS classification system identified three distinguishable collagen patterns in the vicinity of the growing tumor colony (Figure 1). The TACS-1 signature is characterized by unorganized fibrils with a wavy appearance located in areas farther from the tumor. The TACS-2 signature shows stretched collagen fibrils aligned parallel to the edge of the tumor cluster. The TACS-3 signature can be identified by collagen fibrils oriented radially from the tumor cluster, which often develop at the site of local cell invasion. Recently, this TACS classification was extended to include large-scale patterns that either span several cell colonies—TACS-4 to TACS-6—or define different ECM fibril patterns within sparsely located cells—TACS-7 and TACS-8. These collagen signatures were used in a large retrospective study to stratify tumor histology images and identify patients with a high risk of breast cancer recurrence [16].

However, all of these histology images, whether from clinical or mouse tumors, represent only one-at-a-time data. Here, we use mathematical modeling to investigate the emergence of TACS patterns around a cell colony and the transitions between these collagen signatures. In contrast to static histology images, our model is able to trace the changes in TACS patterns dynamically, both in space and time. To our knowledge, this is the first mathematical model to address the formation of various TACSs and transitions among them. The ECM structure and interactions between individual cells and ECM fibrils are best captured by agent-based models (ABMs). ABMs are capable of reproducing tissue heterogeneities and diverse morphologies, such as mammary ducts or multi-cellular organoids [17,18]. In particular, the off-lattice models that we and others have developed [19,20,21] can also incorporate cell and ECM mechanics. In the present study, we coupled our multi-cellular lattice-free (*MultiCell-LF*) model [21,22,23,24,25] with the vector field representation of the ECM fibril structure. This approach was inspired by the model of fibroblast–ECM interactions during wound healing developed by Dallon and colleagues [26,27,28,29]. However, our model is used for a different application: ECM remodeling by growing and migrating cells. Among other models of the interplay between cells and ECM fibril structures are the beam/truss elements to represent individual fibers, and spherical elements to model single cells or cell doublets [30,31,32,33,34], growing multi-cellular spheroids [35,36], colonies of fibroblasts [37], or two-scale frameworks using the microscopic fiber phase [38,39]. The Cellular Potts model has been used to represent both deformable tumor cells and remodeled ECM fibers in angiogenesis [40], tumor cell invasion [41], and the durotaxis process [42]. In the present study, we will comprehensively analyze the emerging patterns of ECM during tumor progression. We will trace the physical interactions between individual cells, such as repulsive or adhesive forces. We will also model the interplay between cells and ECM fibrils, such as bundle formation or changes in fibril orientation and stiffness.

The rest of the paper is organized as follows. First, the mathematical model is presented in detail in Section 2. Next, we will discuss the algorithm for ECM remodeling using an example of a single cell migrating through the ECM in Section 3.1. This is followed by simulations of the emerging patterns of TACS-1 in Section 3.2, TACS-2 in Section 3.3, and TACS-3 in Section 3.4. Finally, we will study the sequential transitions among TACSs in Section 3.5. We will close the paper with a discussion in Section 4.

## 2. Materials and Methods

In this section, we rigorously describe the mathematical equations that define our model. In our approach, all cells are modeled as individual agents, and ECM fibrils are represented by a discrete vector field with a specified fibril direction and stiffness value for each fibril bundle. All physical interactions between cells and fibrils are modeled using spring forces that act locally within a small neighborhood of each cell. This is biologically relevant since cells are independent entities that can act in a collective fashion, and the ECM is characterized by fibril orientation, as well as by physical density or stiffness.

### 2.1. Cell–ECM Interactions

The individual cells and ECM fibrils interact reciprocally in our model. A compliant ECM can be remodeled by migrating or growing cells, while a stiff ECM can impact the direction of a nearby motile cell. The cells that are passively relocated during tumor growth can push on the neighboring ECM fibrils. As a result, the cells can change their fibril orientation due to the force exerted by the cell. Fibril stiffness can also be increased depending on the distance from the pushing cell. Elevated ECM stiffness can, in turn, trigger a cell’s behavior. The cells can start pulling on the fibers and then migrate along their direction. This affects the fibril stiffness and orientation due to the force exerted by the migrating cell. The equations governing these cell behaviors and cell–ECM interactions are defined below.

### 2.2. Cellular Component of the Model

Each cell is capable of proliferation and migration. The direction of a motile cell depends on two factors: contact guidance with nearby fibrils and a prescribed persistent movement. We can specify in each simulation which movement component is more prevalent. Moreover, the nearby cells can either adhere to or push on one another by exerting appropriate forces.

Each tumor cell is represented by the position of its nucleus $X_{i}$, current age $A_{i}$, maturation age $A_{i}^{mat}$, and a constant radius R. When the cell reaches its maturation age, is not overcrowded, and is not growth-arrested (as described in Section 2.4), it will divide and produce two daughter cells with the following coordinates:

(1) $$X_{i_{1}}=X_{i}+0.5\cdot R(cos\theta ,sin\theta )\;\;\;\;and\;X_{i_{2}}=X_{i}-0.5\cdot R(cos\theta ,sin\theta ),\;$$

where θ∈[0, 2π] is a random angle. Cell overcrowding means that there are more than 12 cells within the neighborhood of a radius equal to two cell diameters. Both daughter cells’ current ages are set to zero, and their maturation ages are inherited from a mother cell, with random fluctuations within ±15%:

(2) $$A_{i_{1}}={\;A}_{i_{2}}=0\;\;\;\;\;and\;\;\;\;\;A_{i_{1}}^{mat},A_{i_{2}}^{mat}=A_{i}^{mat}\pm \;\tau \;\;\;with\;\tau \in [-0.15,0.15]\cdot A_{i}^{mat}.$$

Since the daughter cells are located half of their common radius apart, their shapes will overlap. To resolve this, repulsive forces are applied to every cell. If $X_{i}$ and $X_{j}$ represent the coordinates of two cells, the repulsive Hookean force $F_{X_{i},X_{j}}^{rep}$ of stiffness $F^{rep}$ exerted on cell $X_{i}$ is defined as follows:

(3) $$F_{X_{i},X_{j}}^{rep}=\left\{\begin{array}{cc} F^{rep}\left(2R-\left\|X_{i}-X_{j}\right\|\right)\frac{X_{i}-X_{j}}{\left\|X_{i}-X_{j}\right\|} & if\;\left\|X_{i}-X_{j}\right\|<2R\\ 0 & otherwise \end{array}.\right.\;$$

It is essential to ensure that the proliferating cells form a compact cell cluster, because upon division, the daughter cells are placed in random directions. Accordingly, adhesive forces are exerted on cells with no more than five neighbors within the radius equal to two cell diameters. If $X_{i}$ and $X_{j}$ represent the coordinates of the two cells, the adhesive Hookean force $F_{X_{i},X_{j}}^{adh}$ of stiffness $F^{adh}$ and the resting length $R^{adh}$ exerted on cell $X_{i}$ is given by the following:

(4) $$F_{X_{i},X_{j}}^{adh}=\left\{\begin{array}{cc} F^{adh}\left(R^{adh}-\left\|X_{i}-X_{j}\right\|\right)\frac{X_{i}-X_{j}}{\left\|X_{i}-X_{j}\right\|} & \;if\;R^{adh}<\left\|X_{i}-X_{j}\right\|<2R^{adh}\\ 0 & \;otherwise \end{array}.\right.$$

Since each cell can be in close proximity to several other cells, the total force $F_{i}$ acting on the cell $X_{i}$ combines the repulsive and adhesive contributions from $N_{R}$ and $N_{A}$ nearby cells, respectively. This total force is given by the following equation:

(5) $${\;\;\;F}_{i}=\sum_{j\neq i}^{N_{R}}F_{X_{i},X_{j}}^{rep}+\sum_{j\neq i}^{N_{A}}F_{X_{i},X_{j}}^{adh}$$

In addition, if the cell is actively migrating (see Section 2.4), a motility force $G_{i}$ is exerted on that cell. The motility force direction is a result of competition between the directions of persistent cell movement $G_{i}^{*}$ and the direction induced by the orientation $h\left(x,y\right)$ of the fibrils that are in contact guidance with the cell, i.e., fibrils located in the cell’s close neighborhood $\chi_{R}$ of radius R. This motility force is defined as follows:

(6) $$\;\;\;G_{i}=G\frac{{\tilde{G}}_{i}}{\left\|{\tilde{G}}_{i}\right\|}\;\;\;\;\;\;with\;\;\;\;{\tilde{G}}_{i}=\alpha G_{i}^{*}+\left(1-\alpha \right)\frac{1}{N_{i}}\sum h\left(x,y\right){\;\chi }_{R+2∆x}\left(\left(x,y\right),X_{i}\right)\;$$

where α is the persistence coefficient, G is the maximal magnitude of the motility force, and $N_{i}$ is the number of fibrils within two grid widths from the cell boundary (R+2∆x). The cell neighborhood $\chi_{R}$ of radius R is defined as follows:

(7) $$\;{\;\chi }_{R}\left(\left(x,y\right),X_{i}\right)=\left\{\begin{array}{ccc} 1 & if & \left\|\left(x,y\right)-X_{i}\right\|\leq R\\ 0 & & otherwise. \end{array}\right.$$

Cell relocation is governed by the overdamped spring equation [22], where ν is the viscosity of the surrounding medium, $G_{i}$ is the cell motility force, and $F_{i}$ is a cumulative repulsive and adhesive force:

(8) $$\;\;\;\;\;\;\frac{dX_{i}}{dt}=\frac{1}{\nu }{(F}_{i}+G_{i})\;$$

### 2.3. Extracellular Component of the Model

The ECM physical properties, both the fibril orientation and stiffness, can be modified by the nearby cells, provided that the ECM is compliant. These modifications are proportional to the distance from the cell: the fibrils located closer to the cell will undergo larger changes than fibrils located 2–3 layers from the cell. The fibril orientation can either become aligned with the direction of the migrating cell pulling on the fibrils, or can create a barrier to the growing cells, which push on the nearby fibrils.

The ECM structure is modeled as a unit vector field h(x,y), which provides the directions of the ECM fibers at a given point (x,y) and a scalar value ξ(x,y) representing the ECM stiffness at that same point. Initially, all fibrils have random directions and uniform stiffness. Subsequently, the fibril’s orientation may be impacted by the nearby cells. However, the extent of ECM remodeling $\tilde{h}\left(x,y\right)$ depends on fibril compliance β to the direction of the force exerted by the growing or moving cells, thus:

(9) $$\tilde{h}\left(x,y\right)=(1-\beta )\;h\left(x,y\right)+\beta \sum g_{i}^{⊥}{\;\chi }_{R+2∆x}\left(\left(x,y\right),X_{i}\right)\;+\beta \sum g_{i}^{∥}{\;\chi }_{R+∆x}\left(\left(x,y\right),X_{i}\right)\;$$

where $g_{i}^{⊥}=⊥F_{i}$ is a vector perpendicular to the direction of a growing cell, since the growing cell pushes on the nearby fibrils; and $g_{i}^{∥}=∥G_{i}$ is a vector parallel to the direction of a migrating cell, since the moving cell can align the fibrils. The summations are over the growing or migrating cells, respectively. The unit vector of the ECM orientation is as follows:

(10) $$h\left(x,y\right)=\frac{\tilde{h}\left(x,y\right)}{\left\|\tilde{h}\left(x,y\right)\right\|}\;\;\;\;$$

Fibril stiffness ξ(x,y) can be increased due to pressure induced by a nearby cell $X_{i},$ which either migrates through the stromal space or is being relocated during tumor growth. Thus, the change in ECM stiffness is defined as follows:

(11) $$\;\;\;\;\frac{d\xi \left(x,y\right)}{dt}=\Delta \xi \cdot \beta \sum_{\;}^{\;}{\;\omega }_{R,\Delta x}\left(\left(x,y\right),X_{i}\right)+\sum_{\;}^{\;}\left(\beta \Delta \xi +{\Delta \zeta_{i}\;\varpi }_{R,\Delta x}\left(\left(x,y\right),X_{i}\right)\right)\;$$

where ${\;\omega }_{R,h}$ and $\varpi_{R,h}$ are the regions of interactions with the migrating or growing cell, respectively. If the cell $X_{i}$ is migrating, it will pull on the nearby fibrils and increase their stiffness by Δξ, subject to the compliance value β. The increase in fibril stiffness is also inversely proportional to the fibril distance from the cell:

(12) $${\;\omega }_{R,h}\left(\left(x,y\right),X\right)=\left\{\begin{array}{cc} 3 & if\;\;\;\;0<\left\|\left(x,y\right)-X\right\|\leq R\;\\ 2 & \;if\;\;\;\;\;\;\;\;\;\;\;\;\;\;\;R<\left\|\left(x,y\right)-X\right\|\leq R+h\sqrt{2}\\ 1 & if\;\;\;\;\;R+h\sqrt{2}<\left\|\left(x,y\right)-X\right\|\leq R+2h\sqrt{2} \end{array}\right.$$

If the cell $X_{i}$ is relocated due to the expanding tumor cluster, the cell will push on the surrounding fibrils and increase their stiffness by Δξ, subject to the compliance value β. In addition, the fibrils located on the grid points enclosed by the cell $X_{i}$ will be redistributed to the surrounding fibril points by increasing their stiffness by $\Delta \zeta_{i}$. This increase in fibril stiffness is also inversely proportional to the distance from the cell center:

(13) $${\;\varpi }_{R,h}\left(\left(x,y\right),X\right)=\left\{\begin{array}{cc} 0\;\;\;\;\;\;\;\;\; & \;\;\;\;\;\;\;\;\;if\;\;\;\;0<\left\|\left(x,y\right)-X\right\|\leq R\;\;\;\;\;\;\;\;\;\;\\ 3\;\;\;\;\;\;\;\; & \;\;\;\;\;\;\;\;\;\;\;\;\;\;\;\;if\;\;\;\;R<\left\|\left(x,y\right)-X\right\|\leq R+h\sqrt{2\;}\;\;\;\\ 2\;\;\;\;\;\;\;\;\; & if\;\;\;\;R+h\sqrt{2}<\left\|\left(x,y\right)-X\right\|\leq R+2h\sqrt{2}\\ 1\;\;\;\;\;\;\;\;\; & if\;\;\;\;R+2h\sqrt{2}<\left\|\left(x,y\right)-X\right\|\leq R+3h\sqrt{2} \end{array}\right.$$

### 2.4. Cell Behavior Modulated by Stiffness of the Nearby ECM

Each cell can sense the stiffness of the ECM fibrils in their vicinity, and certain cell behaviors can be regulated based on that information. For example, the ECM can create a barrier to cell proliferation if its stiffness is elevated and the orientation is perpendicular to that cell. However, increased ECM stiffness can also induce cell migration. In order to make a decision about proliferation or migration, the cells must be able to determine the stiffness of the nearby ECM. This process of ECM sensing is described below.

The local cumulative fibril stiffness $\Xi_{i}$ sensed by the cell $X_{i}$ from the neighborhood of radius R+2∆x is defined as follows:

(14) $$\;\;\;\Xi_{i}=\sum_{\;}^{\;}\xi (x,y)\;{\;\chi }_{R+2∆x}\left(\left(x,y\right),X_{i}\right)$$

The locally sensed fibril stiffness can act as a barrier for a cell and result in cell growth arrest, if $\Xi_{i}>\Xi^{div}$. On the other hand, elevated fibril stiffness can induce cell migration, if $\Xi_{i}>\Xi^{mot}$.

### 2.5. Model Parameterization

The model parameterization was based on experimental measurements and values reported in the scientific literature. We used a tumor cell radius of R=8 μm [43] and a maturation age of $A_{i}^{mat}=20\;h\pm 15\%$ [43]. The repulsive and adhesive force stiffnesses were $F^{rep}=50\;\mu g/\mu m\cdot s^{2}$ and $F^{adh}=5\;\mu g/\mu m\cdot s^{2}$, respectively [44]. The medium viscosity was ν=250 μg/μm·s [45]. The ECM grid width of ∆x=4 μm represents a bundle of fibrils. Initially, we considered a uniform stiffness of $\xi_{0}=1\sigma \;(=100\;Pa$ after scaling, $\sigma =10^{5}\;\mu g/\mu m\cdot s^{2})$, which is characteristic of a normal mammary tissue [46]; this ECM stiffness could be increased at a rate ∆ξ=0.00004σ every time step, potentially, up to a cancerous level of $\xi_{max}=5\;kPa\;(=500\sigma )$ [46]. The maximal magnitude of the motility force was assumed to be $G=0.08\;\mu g\cdot \mu m/s^{2}$, which results in a cell speed of 0.00032 μm/s, for the cell that exerts only the persistent movement. The persistent direction of cell migration $G_{i}^{*}$ was varied in the simulations described below, as were the values of the fibril compliance coefficient β, and the persistent migration coefficient α. A time step of Δt=2.5 s was used in all simulations.

## 3. Results

Our goal here was to identify the rules of cell–ECM interactions that guide the development of various patterns of alignment of the ECM fibrils in the vicinity of a developing colony of tumor cells. We used the off-lattice hybrid agent-based model, *MultiCell-LF*, in our simulations. First, as described in Section 3.1, we simulated the migration of a single tumor cell that remodeled the nearby ECM. This was carried out to illustrate how our model works. Subsequently, we investigated the rules that allowed for the emergence of three different tumor-associated collagen signatures: TACS-1 in Section 3.2, TACS-2 in Section 3.3, and TACS-3 in Section 3.4. Finally, the sequential transitions among the TACS patterns were simulated and are presented in Section 3.5.

### 3.1. ECM Remodeling by a Single Migrating Cell

In this section, we present four simulations that illustrate cell–ECM interactions. The direction of cell movement is a combination of persistent movement toward the top-left corner and contact guidance, where a cell follows the direction of the nearby ECM fibers. On the other hand, the degree of ECM remodeling depends on its compliance. For the compliant ECM, the fibrils’ orientation will be remodeled in the direction of the migrating cell. Otherwise, the fibrils will retain their orientation.

**Figure 2:**
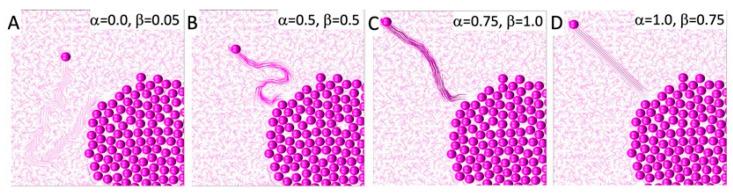
ECM remodeling by migrating cells. A combination of persistent movement toward the left-top corner and contact guidance with the nearby fibril orientation. The traces left depend on ECM compliance. Color shades correspond to different ECM fibril stiffnesses. (**A**) Persistent migration coefficient α=0 and compliance coefficient β=0.05; (**B**) α=0.5, β=0.5; (**C**) α=0.75, β=1; (**D**) α=1, β=0.75.

The direction of persistent migration in all simulations was set to $G_{i}^{*}=\left[-1,1\right]/\sqrt{2}$, so the cell’s goal was to reach the upper-left corner of the domain. However, the actual cell movement is also influenced by the orientation of the fibrils located in the cell’s vicinity, according to Equation (6). The relationship between a cell’s persistent movement and movement due to contact guidance from the surrounding fibrils is defined by the persistent migration coefficient α. The examples presented in Figure 2 show four different cell behaviors. In Figure 2A, the cell entirely follows the fibril orientation since the persistent migration coefficient is α=0. In Figure 2B,C, persistent migration influences the cell direction to an increasing degree, with α=0.5 and α=0.75, respectively. In the final example shown in Figure 2D, the cell ignores the fibril orientation and moves strictly in the pre-defined persistent movement direction.

Additionally, the migrating cell can modulate the orientation of the nearby fibrils if they are compliant, according to Equation (9), as well as the fibril stiffness, according to Equation (12). This is defined by the compliance coefficient β. The four examples presented in Figure 2 showcase ECM with different remodeling capabilities: β=0.05, 0.5, 1, and 0.75 in Figure 2A–D, respectively. Increased fibril stiffness is also reflected in the fibril colors, with darker colors representing stiffer fibrils. However, it is worth noting that if the cell migrates quickly, the fibril stiffness does not increase as much as it does when the cell wanders through the tissue for a longer period of time by following the fiber direction. This is shown in Figure 2D, where β= 0.75, and in Figure 2B, where β= 0.5. Therefore, taken together, Figure 2 presents cases where the cell path towards the upper-left corner is tortuous, because it follows the fibers with their random orientations, which remain relatively unchanged, as well as cases where a cell moves straight to the corner in a persistent fashion and leaves behind a highly remodeled ECM. In these illustrative examples, we assumed that once the ECM was remodeled by the cell, it retained its new orientation and stiffness, so these effects would be visible at the end of the simulation. In reality, there may be some elasticity effects that cause the ECM fibrils to return to their initial configuration. Such a scenario is not modeled here.

### 3.2. Formation of TACS-1

**Figure 3:**
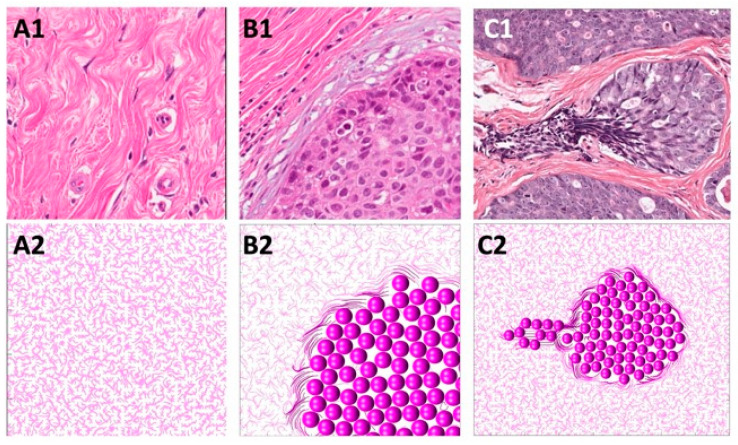
Simulated models of TACSs. (**A1**) The wavy, unorganized ECM pattern of TACS-1 from a histology of normal mammary tissue. (**A2**) Simulated ECM with randomly aligned fibrils as a model of TACS-1. (**B1**) ECM patterns of TACS-2 encircling the cell cluster. (**B2**) Simulated ECM with fibrils aligned parallel to the cell cluster as a model of TACS-2. (**C1**) ECM pattern of TACS-3 aligned near the invading cells; (**C2**) Simulated ECM with fibrils pointing radially outward from the tumor at the side of the invasion; model of TACS-3. The increased fibril stiffness is indicated by darker colors.

In this section, we present how the normal ECM structure is defined in our mathematical model. Normal breast tissue histology comprises ductal structures that are surrounded by loose stromal connective tissue [47,48,49]. This unorganized pattern of ECM fibrils can be observed experimentally with second-harmonic generation imaging or real-time polarization microscopy [47,49,50] in regions located far from the growing tumor cell colonies and is referred to as tumor-associated collagen signature 1 (TACS-1). TACS-1 is shown in Figure 3(A1) (H&E staining), where no defined arrangement of ECM fibrils is visible. Upon tumor development, collagen density increases locally due to deposition by activated stromal cells [10,13].

The properties of the normal ECM are mathematically modeled as a unit vector field h of random orientation: $h=\left[\varepsilon_{x},\varepsilon_{y}\right]/\sqrt{\varepsilon_{x}^{2}+\varepsilon_{y}^{2}}$, where $\varepsilon_{x},\varepsilon_{y}\in [-1,1]$ are random numbers. The ECM fibril stiffness is uniformly low: ξ=1σ. This computationally simulated pattern is shown in Figure 3(A2).

### 3.3. Formation of TACS-2

In this section, we identify the rules of cell–ECM interactions that will allow us to recreate the TACS-2 fibril pattern in the vicinity of the growing cell colony. During tumor growth, the expanding cell colony can impose pressure on the surrounding ECM fibrils, rearrange their orientation, and increase their stiffness. As a result, the elongated and straightened collagen fibrils become aligned parallel to the tumor–stroma boundary and encapsulate the tumor cell cluster [49,51]. This ECM fibril pattern is shown in Figure 3(B1) (H&E staining) and is known as tumor-associated collagen signature 2 (TACS-2).

In the mathematical model, the proliferating cells push on their neighboring cells and, as a result, push off the surrounding fibrils. This affects the fibrils’ orientation by aligning them perpendicularly to the cell relocation force of $g_{i}^{⊥}=⊥F_{i}$. For the larger compliance coefficient β in Equation (9), this leads to ECM alignment along the cluster boundary shown in Figure 3(B2). Moreover, every time the growing cell pushes on the fibrils, their stiffness at the point of contact with the cell increases according to Equation (13). This is shown by the fibers of darker colors in Figure 3(B2).

**Figure 4:**
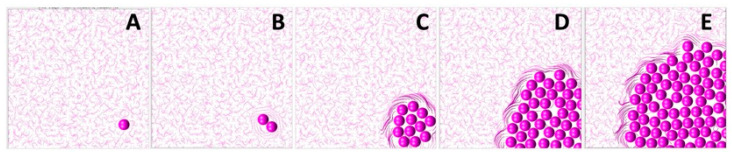
Development of TACS-2 in the mathematical model. (**A**–**E**) Snapshots from a simulation showing the emergence of ECM fibrils aligned parallel to the boundary of the growing cell cluster and encapsulating it at times 0, 1, 3, 5, and 6 days, respectively. The increase in local ECM fibril stiffness is shown by different colors.

The development of TACS-2 in the mathematical model is illustrated in more detail in Figure 4. The simulation starts in Figure 4A with an individual cell located in the non-rigid randomly oriented ECM fibril structure. Upon division, the shapes of both daughter cells overlap, and they must push on one another to resolve that. During this process, they push on the surrounding ECM fibrils. Since the fibrils’ compliance coefficient is β=0.5, they exert resistance and realign perpendicularly to the pushing cells, as shown in Figure 4B. This, in turn, increases their stiffness. This is shown by different colors in Figure 4. The process continues as the cluster of tumor cells grows in size, and more fibrils are relocated by the pushing cells. As presented in Figure 4C–E, only fibrils in the close vicinity of the tumor border are remodeled. Those located far from the tumor remain randomly oriented and with no increase in their stiffness.

### 3.4. Formation of TACS-3

In this section, we propose the rules for cell–ECM interactions that will lead to the development of the TACS-2 fibril pattern near the migrating cells. During the emergence of tumor invasion, the ECM fibrils are primarily aligned in the direction perpendicular to the tumor boundary, forming straightened bundles that point radially outward from the cell cluster. This ECM pattern, known as tumor-associated collagen signature 3 (TACS-3), is shown in Figure 3(C1) (H&E staining). TACS-3 has also been observed as accompanying cell cohorts migrating along the fibrils, thus facilitating cell invasion [12,13,49].

The mathematical algorithm used to achieve this pattern requires that the migrating cells pull on the nearby fibrils and change the fibril orientation to become parallel to the cell motility force of $g_{i}^{∥}=∥G_{i}$. At the same time, fibril stiffness also increases at the point of contact with the migrating cells. In Figure 3(C2), the direction of persistent migration was chosen to be $G_{i}^{*}=\left[-1,0\right]$, so the cells would move horizontally to the left. Since the compliance coefficient β in Equation (9) is larger, the ECM can also become aligned in the direction of the migrating cells and perpendicular to the tumor boundary. In addition, every time the fibril orientation changes due to the cells pulling on the fibrils, the stiffness of that particular ECM bundle increases according to Equation (12). This is indicated in Figure 3(C2) by fibers of darker colors.

**Figure 5:**
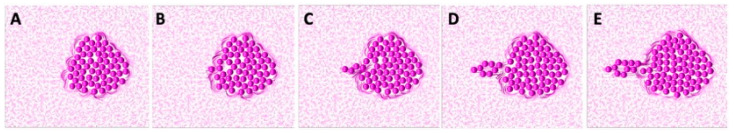
Development of TACS-3 in the mathematical model. (**A**–**E**) Snapshots from a simulation showing the emergence of tumor cell invasion at times 0, 14, 30, 50, and 72 h. The ECM fibrils become aligned in parallel to the migrating cell cohort in the direction radial outward from the cell cluster. The local ECM fibril stiffness is shown in different colors.

A more detailed development of TACS-3 is presented in Figure 5. The starting configuration shown in Figure 5A is a cluster of cells residing in the ECM with random fibril orientations everywhere, except near the tumor boundary. Upon initiation of the invasion process, a single tumor cell follows the persistent movement direction. Since the value of the persistent migration coefficient is large, α=1, cell migration is not influenced by the local orientation of the ECM fibrils. The cell thus migrates horizontally, as shown in Figure 5B,C. Since the ECM compliance coefficient is also large, β=0.75, the migrating cell remodels the nearby ECM fibrils by aligning them in the same direction. This is shown in Figure 5C. Subsequently, the other cells follow the leader cell and exert pulling forces on the nearby fibrils, leaving behind a wide band of horizontally aligned ECM. These fibrils have increased stiffness, as indicated by the darker colors in Figure 5D,E. In this simulation, there were sporadic cell divisions if the migrating cells left free space behind. As in the case of the TACS-2, only the fibrils in the close vicinity of the migrating cells were remodeled. The other fibrils remained randomly oriented, with unchanged stiffness.

### 3.5. Transitions between TACS Patterns

In this section, we identify the rules of cell–ECM interactions that allow for a dynamic transition from TACS-1 to TACS-2, and then to TACS-3 in the same simulation. These three different TACS patterns have been observed in experimentally grown tumors [12,13,49] and in histology images from clinical tumor samples [16,50,52]. Usually, these images represent only data from one snapshot at a time. Thus, in order to infer how one TACS pattern can change into another, one must collect images from different tissues and rely on averaged values. With computational simulations, we are able to trace TACS transitions longitudinally in the same in silico tissue.

**Figure 6:**
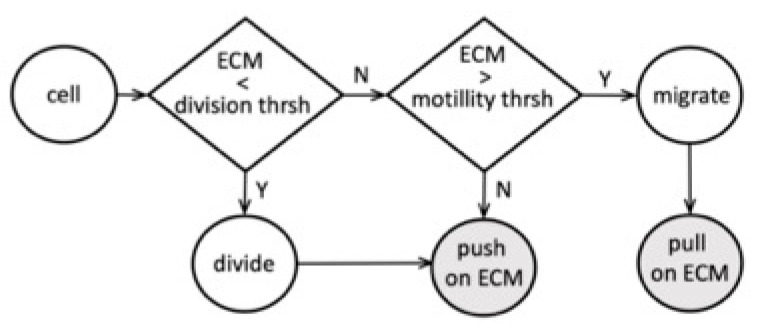
Schematics of tumor cell–ECM interactions of the sequential transitions among TACS patterns.

We examined the interactions between tumor cells and the surrounding ECM and determined the model parameter thresholds that allow for the sequential emergence of different TACS patterns. A flowchart of the proposed cell–ECM interactions is shown in Figure 6. In our model, the tumor cell can react to the ECM stiffness sensed from its vicinity. Based on this information, the cell can either divide if the total sensed stiffness is below the prescribed division threshold or become growth-arrested if the sensed stiffness exceeds that threshold. The dividing cell then pushes on the surrounding fibrils and remodels their orientation and stiffness. These changes in the ECM depend on the parameter values of persistence α and compliance β. If the cell does not actively divide, it can become motile, provided that the surrounding ECM fibril stiffness exceeds the prescribed motility threshold. The migrating cell then pulls on the nearby fibrils and remodels them. The non-dividing and non-motile cells can still push on the surrounding fibrils if it is itself pushed by the nearby proliferating cells.

**Figure 7:**
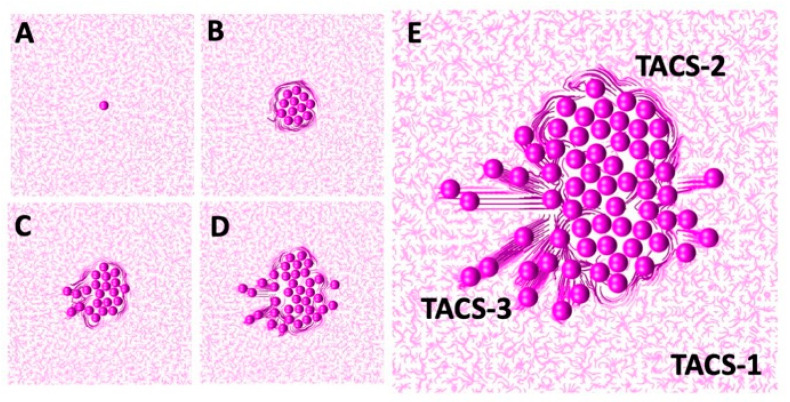
Dynamic development of TACS patterns. (**A**) An initial single cell and TACS-1; (**B**) TACS-2 around a small cell cluster; (**C**,**D**) The migrating cells with TACS-3; (**E**) All three co-existing patterns. Snapshots taken on days 0, 3, 4, 5, and 6. Fibril stiffness is shown by different colors.

The successful sequential development of all three TACS fibril patterns is shown in Figure 7. An initial single cell was embedded in the randomly oriented ECM, displaying the TACS-1 pattern, as shown in Figure 7A. Due to the multiple cell divisions, a small cluster of cells formed. The growing cells pushed on the surrounding ECM fibrils, which generated the TACS-2 pattern shown in Figure 7B. During this process, fibril stiffness also increased, which is indicated by the darker colors of ECM fibrils near the cell cluster. The ECM reached the growth arrest threshold around the top part of the cluster and the cell migration threshold on the left side. Since the growth-arrested cells were no longer dividing, the surrounding fibril stiffness remained below the migration threshold until the end of this simulation. However, the cells that sensed ECM stiffness above the migration threshold became motile. As a result, the migrating cells created the TACS-3 fibril pattern shown in Figure 7C,D. The final cell configuration showing all three TACS patterns co-existing in the same growing tumor cell colony is presented in Figure 7E. The following parameters were used in this simulation: the threshold for cell growth arrest $\Xi^{div}=30\;\sigma$, the threshold for initiation of cell migration $\Xi^{mot}=50\;\sigma$, the compliance coefficient β=0.5, and the persistent movement coefficient α=1; the persistent direction of the motile cells was outward from the center of the computational domain.

**Figure 8:**
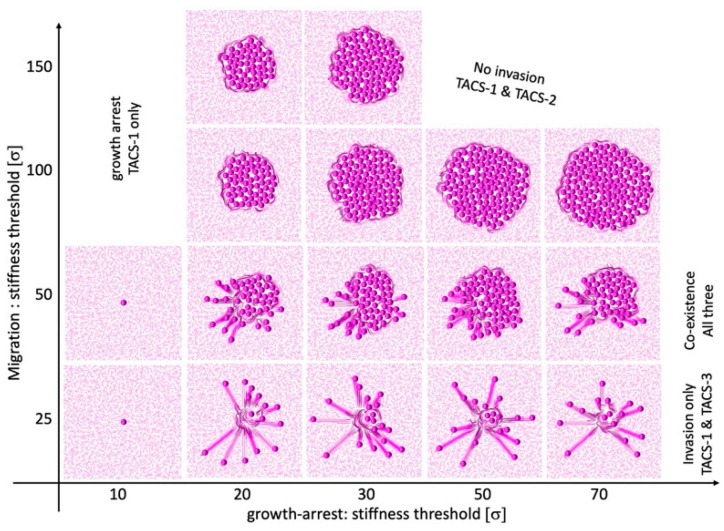
Classification into different TACS patterns for combinations of local stiffness thresholds. A chart showing the emergent TACS patterns of the specified thresholds of local ECM stiffness, which results either in cell growth arrest or in the initiation of cell migration. The domination of one or two TACS patterns, or the coexistence of all three is indicated.

The emergence and possible co-existence of all three TACS patterns depends on the combination of thresholds for cell growth arrest $\Xi^{div}$ and cell migration $\Xi^{mot}$, which can either initiate or suppress a given cell process. Figure 8 shows the parameter space of our mathematical model, which classifies the simulation outcomes according to the generated TACS patterns. In general, the very low threshold for growth arrest results in total growth suppression and no ECM remodeling. As a consequence, the only ECM pattern present is TACS-1. This is shown in the left column of the chart in Figure 8. 

The low motility threshold leads to cell spread, with no colony formation. This is accompanied by the presence of TACS-1 and TACS-3 patterns. The corresponding cellular configurations are shown in the bottom row of the chart in Figure 8. In contrast, the large motility threshold results in large colony formation with no cell invasion and only the TACS-1 and TACS-2 patterns of the ECM. These cellular colonies are shown in the two top rows of the chart in Figure 8. In our model, the co-existence of all three TACS fibril patterns only emerged for moderate motility and growth arrest thresholds. This is shown in the middle row of the chart in Figure 8.

The TACS chart in Figure 8 can be used for comparison with experimental or clinical imaging data. One can map a TACS pattern observed in biological data onto the TACS chart to predict which changes in local ECM stiffness lead to the emergence of a given pattern. Alternatively, one can use this chart to identify the growth arrest and migration thresholds when designing laboratory experiments. This approach is similar to the Morphochart technique, which we previously developed for mammary acini [53,54].

## 4. Discussion

In this paper, we used an off-lattice hybrid agent-based Multi-Cellular Lattice-Free (*MultiCell-LF*) model to investigate the cell–ECM physical interactions that lead to the emergence of various ECM fibril alignments. It has been observed experimentally and in clinical samples of breast cancers that three specific TACS are characteristic of distinct stages of cancer progression [7,12,13]. The TACS-1 signature, which has a wavy appearance with unorganized fibrils, was detected in areas located farther from the growing tumor cell colony. The TACS-2 signature was characterized by ECM fibrils aligned parallel to the edge of the developing tumor cluster. Finally, the TACS-3 signature was found to exhibit ECM fibrils oriented radially from the tumor cluster at the sides of the tumor cell invasion. Using our mathematical model, we identified the rules for the cell–ECM physical interactions that resulted in the given fibril alignment. Our starting point was the TACS-1 signature with randomly oriented ECM fibrils and uniformly low stiffness. For the TACS-2 and TACS-3 patterns, the fibril orientation and stiffness were rearranged by dividing and/or migrating cells and depended on the chosen growth arrest and migration thresholds, as well as the ECM compliance parameter. The model outcomes were visualized using in-house MATLAB^®^ (v. 2022a) routines, which utilized the *curvvec.m* function [55] for drawing the ECM fibril structure.

The fibril stiffness thresholds identified by our model are biologically relevant. It has been experimentally demonstrated that ECM stiffness elevated 10-fold or more in comparison to normal tissue stiffness, was able to induce invasive behavior in the multi-cellular spheroids derived from a nontumorigenic epithelial cell line [56,57,58]. It has also been shown that the increased stiffness of the ECM can encapsulate the tumor and prevent invasion in the early stages of tumor development [59]. However, when ECM stiffening is initiated after cell invasion, cancer cell migration will be promoted [59]. The generated cellular behaviors are also comparable to the experimental results [59,60]. For example, highly invasive cells can spread radially outward from initial seeding positions. In contrast, cells with a lower invasive potential or those in confined extracellular microenvironments can generate small cell cohorts that protrude together through the surrounding ECM. Moreover, cells with low migratory potential can become encapsulated with no visible microinvasions.

Computational models, like the one we have developed here, are capable of tracing longitudinal changes in the same modeled organism, which is not always possible in laboratory experiments. We have shown here how TACS patterns can progressively emerge in the same in silico tissue patch and dynamically evolve from one collagen signature to another. To our knowledge, this is the first mathematical model that addresses TACS formation and its dynamic transformations. Moreover, we propose here that a combination of feedforward and feedback loops, which enable switching between cell growth with ECM pushing and cell migration with ECM pulling, together form a mechanism for TACS development. These predictions require further experimental validation.

We considered here only the mechanical interactions between cells and ECM, and the physical properties of fibril orientation and stiffness. This is a simplification of the real process, which involves fibril alignment, increased fibril density, fibril cross-linking, and elevated ECM stiffness. This simplification was performed to reduce the number of model parameters. However, these additional functional relationships could be incorporated into our model and studied in more detail. Further research should also consider the addition of cell–ECM biochemical interactions, such as the secretion of MMPs, which are responsible for ECM degradation and remodeling, and which may result in the removal of migration barriers [6]. Moreover, we focused on modeling the direct interactions between the cell colony and the surrounding ECM. Thus, we neglected potential heterogeneities in the initial ECM stiffness and changes in collagen properties due to external factors, such as the deposition of ECM by fibroblasts. We also did not include in the model other stromal cells, such as immune cells, adipocytes, or fibroblasts. These features can be incorporated into future model extensions. Another aspect to incorporate into our model is the link between cell metabolism and ECM remodeling, as it has been shown that oxygenation and acidity can modulate collagen production and remodeling [61,62,63]. Our study was performed in the context of tumor growth, but this model can be extended to non-tumorigenic cells interacting with the ECM. However, this will require model re-calibration.

The developed mathematical model allows for the longitudinal tracing of changes in ECM organization around growing tumors. To this point, we incorporated into this model only mechanical interactions between the ECM and the cells embedded in it. However, after accounting for the factors described above, the model could be used to stratify the differences in the stroma surrounding breast cancer lesions, including normal, desmoplastic, ductal carcinoma in situ, and ductal cancer with microinvasions. With computational simulations of transitions between different ECM signatures, like the TACS, we could determine the likelihood of the emergence of tumor microinvasions, which are the first step to cancer invasion. This will help advance cancer diagnostics, and the prognosis of tumor progression and may serve as a histology-based biomarker.

## Data Availability

The developed mathematical model code is available at the GitHub site: http://github.com/rejniaklab/silicoECM since the publication date of this manuscript under GNU General Public License v3.0.
